# Frailty as a Surgical Vital Sign: A Narrative Review of Risk Stratification and Outcomes in General Surgery

**DOI:** 10.7759/cureus.108188

**Published:** 2026-05-03

**Authors:** Ambrose Loc T Ngo, Matthew Tiojanco, Ian Miller, Linda Nguyen, Uyen Tam Nguyen, Carolyn Duong

**Affiliations:** 1 College of Osteopathic Medicine, Kansas City University of Medicine and Biosciences, Kansas City, USA; 2 College of Pharmacy, Western University of Health Sciences, Pomona, USA

**Keywords:** clinical frailty scale, frailty, frailty index, general surgery, length of stay, mortality, perioperative risk, postoperative complications, risk stratification, surgical outcomes

## Abstract

Frailty has emerged as a critical determinant of perioperative risk, yet it remains underutilized in routine surgical assessment. As the global population ages, an increasing proportion of patients undergoing general surgery present with diminished physiologic reserve that is not adequately captured by traditional risk stratification tools. This narrative review examines frailty as a multidimensional construct and its role as a prognostic marker in general surgery, with the aim of synthesizing current evidence on its biological basis, evaluating commonly used frailty assessment tools, and examining its impact on postoperative outcomes, including morbidity, mortality, length of stay, and discharge disposition. A comprehensive literature search was conducted using PubMed and Google Scholar to identify relevant peer-reviewed studies published between 2005 and 2025, with an emphasis on surgical populations and clinically validated frailty assessment tools. Across surgical populations, frailty demonstrates strong independent associations with adverse outcomes and has been reported in multiple studies to outperform chronological age and conventional metrics such as the American Society of Anesthesiologists (ASA) classification or comorbidity burden. Among the tools evaluated, instruments such as the Clinical Frailty Scale and Risk Analysis Index demonstrate feasibility for integration into perioperative workflows, while more comprehensive instruments provide additional physiologic granularity when resources permit. Despite variability in assessment methodologies, the collective evidence supports frailty as a robust predictor of surgical vulnerability. Despite variability in assessment methodologies, the collective evidence supports frailty as a robust predictor of surgical vulnerability. Incorporating frailty into routine preoperative evaluation may enhance risk stratification, inform shared decision-making, and guide targeted perioperative optimization strategies. Future efforts should focus on standardization of screening protocols, integration into existing risk calculators, and prospective evaluation of frailty-guided interventions.

## Introduction and background

The burden of postoperative complications in general surgery remains substantial, particularly among older and medically complex patients. With demographic shifts toward an aging population, surgeons are increasingly encountering individuals whose physiologic resilience cannot be adequately characterized by chronological age alone [1,2]. Traditional risk stratification models, including the American Society of Anesthesiologists (ASA) classification and comorbidity indices, provide valuable information but may not fully capture the nuanced interplay between functional decline, physiologic reserve, and vulnerability to surgical stress [3,4]. Nevertheless, the ASA classification remains a widely used system for preoperative risk assessment [4].

Frailty has emerged as a distinct clinical syndrome that addresses this gap. Defined by a reduction in physiologic reserve across multiple organ systems, frailty reflects a diminished capacity to maintain homeostasis in response to stressors such as surgery, infection, or trauma [5]. Importantly, frailty exists independently of age, comorbidity, and disability, although overlap is common [6]. Growing evidence across surgical subspecialties, including colorectal, hepatobiliary, and emergency general surgery, demonstrates that frailty is a robust predictor of postoperative outcomes. Patients identified as frail are more likely to have complications, prolonged recovery, institutional discharge, and mortality compared to non-frail counterparts [7]. Despite this, frailty assessment has not yet been universally adopted in routine surgical practice.

This narrative review aims to define the biological framework of frailty, evaluate commonly used frailty assessment tools in general surgery, and examine the impact of frailty on perioperative outcomes. Furthermore, we explore the emerging paradigm of frailty as a “surgical vital sign” and its implications for modern surgical care.

## Review

Methods

This study is a narrative review conducted using a comprehensive literature search to evaluate the role of frailty in general surgery. The objective was to synthesize evidence regarding frailty as a prognostic marker, characterize available assessment tools, and assess its impact on perioperative outcomes. As such, no quantitative synthesis (e.g., meta-analysis or pooled effect estimates) was performed. A literature search was performed using PubMed and Google Scholar to identify relevant peer-reviewed studies published between 2005 and 2025. Search terms included combinations of “frailty,” “general surgery,” “perioperative risk,” “frailty index,” “clinical frailty scale,” and “surgical outcomes.” Additional references were identified through citation tracking of key articles.

Titles and abstracts were reviewed for relevance, followed by full-text evaluation. Studies were included if they evaluated frailty in adult surgical populations and reported clinically relevant outcomes, including morbidity, mortality, length of stay, or discharge disposition. Both prospective and retrospective cohort studies, meta-analyses, and landmark validation studies of frailty assessment tools were considered. Studies were excluded if they were not in English, were case reports or editorials, or lacked outcome data relevant to surgical populations. A total of approximately 120 articles were initially identified, of which 34 were included in the final qualitative synthesis. Given the narrative design, study selection prioritized clinical relevance and methodological rigor rather than strict adherence to systematic review protocols. Formal systematic review guidelines, such as the Preferred Reporting Items for Systematic Reviews and Meta-Analyses (PRISMA) or PRISMA extension for Scoping Reviews (PRISMA-ScR), were not applied.

Data extraction focused on three primary domains: (1) definitions and pathophysiology of frailty, (2) characteristics and performance of frailty assessment tools, and (3) associations between frailty and postoperative outcomes. Findings were synthesized qualitatively to provide a comprehensive and clinically applicable overview of the literature.

Understanding frailty: definition and pathophysiology

What Is Frailty?

Frailty is a common, complex multidimensional syndrome, particularly in older adults. It is characterized by reduced physiologic reserve and increased vulnerability to stressors; thus, individuals with frailty have a decreased ability to maintain homeostasis in the face of illness and injury [8]. This reduced reserve can be seen across multiple organ systems, such as the musculoskeletal, cardiovascular, pulmonary, and neurologic systems. The resulting effect is that even minor stressors such as infections or changes in medication regimen can lead to clinically functional decline, further complications, and a longer recovery period [8]. Frailty is considered distinct from comorbidity and disability [8]. Comorbidity refers to the presence of multiple diseases that are chronic, while disability refers to limitations of activities of daily living. Frailty, in contrast, is a state of physiologic vulnerability that can exist without disability or comorbidity [8,9]. Although there is overlap among the three, frailty may be classified as its own distinct syndrome [9]. One of the most common frailty screening tools that is used today is the Fried Frailty Phenotype scale [10]. Using this scale, clinicians rate patients based on the presence or absence of five different symptoms: unintentional weight loss, exhaustion, decreased grip strength, slow walking speed, and low physical activity [10].

Biological Basis of Frailty

The biological basis of frailty involves multiple factors and physiological changes associated with the normal aging process. One of the key changes is a process known as inflammaging, which refers to chronic low-grade inflammation, in which elevated inflammatory markers contribute to tissue damage, muscle catabolism, and impaired immune responses to stressors [11]. This mechanism is sometimes referred to as inflammaging. The mechanism of this process includes factors such as obesity, oxidative stress, and inadequate regulation of the immune response [11]. Another component is sarcopenia, which is the loss of skeletal muscle and strength. Sarcopenia leads to decreased mobility and can increase the risk of falls [12]. In addition to the musculoskeletal system, the nervous system, in particular, can become affected with aging, specifically the prefrontal cortex and cerebellum [12]. The neuroendocrine system is another source that contributes to frailty. Sleep, regulation of the hypothalamic-pituitary-adrenal (HPA) axis, and protein catabolism interplay play important roles in the biological processes that lead to frailty [13]. Growth hormone, cortisol, and sex hormones all have an impact on muscle buildup and breakdown and can be dysregulated in various sleep disorders and sleep patterns more commonly seen in the elderly population [13].

Frailty assessment tools in general surgery

Fried Frailty Phenotype

The Fried Frailty Phenotype is one of the most established ways to measure frailty in a clinical setting. It defines frailty as a physical syndrome while classifying patients based on measuring five separate criteria: unintentional weight loss, self-reported exhaustion, decreased grip strength, slowed gait speed, and low levels of physical activity [9]. Patients meeting three or more of these criteria are considered frail, while those with one or two findings fall into the prefrail category [9].

This model has been applied across multiple populations undergoing surgery and has been consistent in showing worse postoperative outcomes [9,14]. In a prospective study of older adults who were undergoing surgery, frailty was independently associated with higher rates of postoperative complications, longer hospital stays, and increased likelihood of discharging to a skilled nursing facility [14]. These findings support the idea that frailty shows an underlying physiologic vulnerability that is often overlooked through the use of traditional risk measures.

Despite the benefits, the Fried model can be difficult to implement in routine preoperative settings. It requires measurement of physical performance, which includes grip strength and gait speed. Measurement of these two actions can take time and may not be feasible in hospital or clinical settings [14]. As a result, while it can be highly beneficial and informative, its use is often limited to research settings or in geriatric assessments.

Clinical Frailty Scale (CFS)

The Clinical Frailty Scale (CFS) is a more practical way of assessing frailty, particularly in clinical environments. It uses a nine-point scale to categorize patients based on overall fitness, functional status, and level of independence [15]. The scale classifies the participants from terminally ill to being very fit [15]. One of the main advantages of the CFS is how quickly it can be applied. It does not require any equipment or formal testing and can usually be completed within a minute based on clinical impression and a brief patient history [15]. Because of this, it has become increasingly common in preoperative clinics and inpatient surgical settings.

Despite its simplicity, the CFS performs well in predicting outcomes. Higher CFS scores have been linked to increased postoperative mortality, complications, and prolonged recovery. This association was found even after adjusting for age and comorbid conditions [16]. In some studies, its predictive ability has been comparable to more complex frailty indexes and scales. The main limitation of the CFS is that it relies on clinician judgment [16]. This can introduce a degree of variability between separate assessors [16]. Despite this barrier, its ease of use and its strong association with outcomes make it a useful screening tool in everyday surgical practice.

Modified Frailty Index (mFI)

The Modified Frailty Index (mFI) defines frailty differently by accumulating the patients' comorbid conditions and functional deficits. Data from the American College of Surgeons National Surgical Quality Improvement Program (NSQIP) database were taken to develop the mFI, which is commonly used in both clinical research and surgical outcomes studies [17]. The mFI has two forms: the original 11-variable version (mFI-11) and a simplified five-variable version (mFI-5). Both of these versions have been shown to correlate with postoperative complications, mortality, and length of stay across many different surgical specialties [17]. The mFI is relatively easy to calculate because the required variables are usually already documented in the medical record and can be applied retrospectively. Despite the convenience of the mFI, it comes with some trade-offs. Because it is largely based on comorbidities, it does not directly measure physical performance or functional decline [17]. This limitation may result in missing patients who appear medically stable but have limited physiologic reserve. This highlights an important distinction between frailty and comorbidity and suggests that the mFI may be most useful when combined with other means of assessment.

Other Tools

In addition to the more commonly used tools, there are many other frailty assessments that have been studied in surgical populations, each with its own strengths and limitations. The Edmonton Frail Scale (EFS) is a multidimensional tool that incorporates cognition, functional status, nutrition, mood, and social support [18]. It gives a broader view of frailty than just physical or comorbidity-based models. EFS is associated with postoperative complications and length of stay, although it does require more time to administer compared to simpler tools [18]. The Risk Analysis Index (RAI) was developed specifically for surgical patients and is designed as a rapid screening tool [19]. Variables like age, comorbidities, and functional status are included and have been shown to predict postoperative mortality with good accuracy. With how quickly it can be completed alongside its surgical focus, it is a practical option for perioperative risk stratification [19].

The Geriatric 8 (G8) is mainly used in oncologic populations and is heavily focused on nutritional status, mobility, and general health [20]. It has been shown to identify more vulnerable patients prior to cancer-related surgery and may help guide preoperative optimization in these patients [20]. With all these tools being considered, there is a lack of a single standardized approach to frailty assessment. While each captures different aspects of vulnerability, their variability also highlights the need for a more unified framework in surgical practice.

Comparison of frailty assessment tools in general surgery

A wide range of frailty assessment tools are currently in use for general surgery: phenotype-based models, clinical judgment-based scales, and deficit accumulation indices. While all strive to identify patients at increased risk of adverse outcomes, they vary in complexity, feasibility, and predictive performance. The Fried Frailty Phenotype represents a phenotype-based approach, which focuses on measurable physical characteristics like grip strength and gait speed [14]. It retains a strong biological grounding and has consistently been shown to predict postoperative complications, longer hospital stays, and discharge to institutional care [14,15]. Despite its effectiveness, its need for physical testing makes it more time-consuming and less practical for day-to-day use in preoperative clinics.

The CFS is a judgment-based tool that focuses on overall functional status and independence. It can be done quickly and at the bedside, which is possible due to the lack of need for specialized equipment [16]. Although comparatively simpler, it has demonstrated strong predictive value for postoperative mortality and functional decline, with its performance showing to be comparable to more complex indices in some studies [16]. Its main limitation is interobserver variability, as scoring depends on the interpretation of each clinician. The mFI has a deficit accumulation approach and is based on comorbid conditions and functional variables coming from large surgical databases [17]. It is easily calculated using routine clinical data and has been validated across many surgical specialties as a predictor of complications and mortality [17]. However, without a direct assessment of physical performance, it may miss some important aspects of physiological reserve.

Other tools, including the EFS, RAI, and G8, offer additional perspectives. The EFS provides a more comprehensive assessment that accounts for cognitive and social factors, but also needs more time to administer [18]. Whereas the RAI was designed specifically for surgical populations and allows for rapid frailty screening while still maintaining strong predictive ability for mortality [19]. The G8 is primarily used in oncologic settings and focuses on nutritional status and overall health, making it especially useful in cancer-related surgery [20]. Overall, no single tool has emerged as the definitive standard for frailty assessment in general surgery. Reported performance metrics vary across studies, and the choice often depends on the clinical context and the balance between accuracy and feasibility. More simple tools like the CFS and RAI are easily integrated into routine workflows, while more comprehensive assessments, such as the Fried phenotype or EFS, provide additional detail when time and resources allow. Regardless of which tool is used, the consistent finding across studies is that frailty is a strong predictor of adverse surgical outcomes.

Impact of frailty on outcomes in general surgery

Postoperative Morbidity

Frailty is associated with increased postoperative morbidity and poor surgical outcomes. Because patients who have frailty have reduced physiologic reserve, as explained previously, they have an impaired ability to respond to the stress of a surgical procedure, which makes them a higher risk for complications associated with the procedure and leads to higher overall postoperative complication rates when compared to their non-frail counterparts [21]. These patients are also more likely to develop surgical site infections (SSIs) as a result of their impaired immune function, leading to poor wound healing [21]. In a meta-analysis of frail versus non-frail patients undergoing total hip and knee arthroplasty, frailty was associated with a higher risk of SSIs, including deep infections such as prosthetic joint infections [22]. Although derived from orthopedic literature, this finding highlights the broader and consistent impact of frailty on postoperative complications across surgical populations. Another source of infection that is higher in frail patients postoperatively is pneumonia. This phenomenon could be due to the decreased pulmonary reserve often observed in frail patients, as well as possibly weakened muscles of the respiratory system [22]. Another factor that could contribute is the longer length of hospitalization after surgeries, which increases the risk of nosocomial infections [23]. An additional complication in frail patients is prolonged postoperative ileus, in which normal bowel motility is decreased after surgery [24]. Together, these complications highlight the importance of identifying frailty in patients and optimizing them before surgery to prevent and reduce postoperative complications.

Mortality

Frailty is an important predictor of mortality in surgical patients. Studies conducted in both ICU and surgical populations have shown that frailty is associated with an increased risk of 30-day mortality, with studies reporting approximately two- to four-fold higher risk compared to non-frail patients [25,26]. This increased risk demonstrates the reduction of physiologic reserve in frail patients and their decreased ability to respond adequately to acute illness and the physical stress of surgery [25,26]. Importantly, frailty remains a strong independent predictor of mortality even after adjustments are made for age. Thus, incorporation of frailty assessment into preoperative evaluation could provide a more accurate understanding of patient risk and help guide decision-making.

Length of Stay and Discharge Disposition

Frailty is also associated with meaningful impacts on the use of hospital resources, a large part of which is driven by longer lengths of stay. Frail patients often have more complicated postoperative courses, which include higher rates of problems like congestive heart failure, and a slower recovery time compared to non-frail patients, which has huge contributions to this observation [27]. These complications and the overall reduced ability of frail patients to respond to stressors also make them more likely candidates to be admitted to the intensive care unit (ICU), delaying recovery and increasing the need for closer monitoring [28]. ICU admission rates can reflect the degree of clinical instability seen in frail patients, requiring more aggressive medical treatments during the postoperative period. As a result, these patients can experience a more complex hospital course and prolonged recovery [28]. After hospitalization, frail patients are also more frequently discharged to skilled nursing facilities and rehabilitation facilities and less likely to be discharged directly home [29]. They often need continued medical care and utilize more resources to recover functionally. Together, these outcomes highlight how frailty affects not only the increased surgical risks but also the complexity of their postoperative course and post-discharge plan.

Emergency General Surgery

Frailty can have major implications in emergency general surgeries. In one study looking at a large group of patients undergoing a wide range of emergency general surgery procedures, including appendectomy, cholecystectomy, and bowel resection, subjects were analyzed on their preoperative and postoperative outcomes and frailty [30]. Patients experienced perioperative complications, with many developing serious postoperative adverse events. These outcomes were found to be worse in patients with higher levels of frailty, who demonstrated increased mortality across all procedures measured [30]. Increasing frailty was consistently associated with higher rates of complications and mortality in a stepwise manner. These patients were also less likely to be discharged directly home after their surgeries [30]. These findings highlight that frailty is a strong predictor of perioperative outcomes and adverse events in emergency general surgery and should be assessed in every patient to help guide decision-making and expectation management for patients and their families.

Oncologic Surgery

In addition to emergency general surgery, frailty has also been shown to have a high impact on oncologic surgeries and cancer treatments. Specifically, in patients undergoing major head and neck oncologic surgeries, frail patients experienced an increased risk of potentially fatal complications that required them to be admitted to the ICU and an increased risk of overall mortality when compared to non-frail patients [31]. Postoperative complications were also found to be higher in these patients, and this was again found to be the case in a step-wise manner [31]. Frail patients handle chemotherapy more poorly than non-frail patients, likely as a result of their already diminished immune response and decreased ability to react and combat physiologic stress. For example, frail patients undergoing chemotherapy can experience higher rates of thrombocytopenia, hyponatremia, and hospitalization rates as a result of the chemotherapy [32]. Decreased tolerance of these medications can also be seen in frail patients, and increased rates of overall disease progression and death have been observed in frail oncologic patients [33].

Frailty as a “surgical vital sign”

Frailty is increasingly being recognized as a key determinant of surgical risk and has been proposed as a “surgical vital sign.” Like traditional vital signs such as blood pressure, heart rate, and oxygen saturation, frailty provides important information about a patient’s physiological reserve and their ability to tolerate stress [14,16]. While vital signs reflect acute physiologic status, frailty shows a more global and longitudinal measurement of vulnerability across multiple organ systems. Frailty can also identify high-risk patients who may not be flagged by traditional measures [14]. Chronological age alone does not reflect physiologic resilience, and commonly used tools like the ASA classification or comorbidity counts can fail to show actual functional decline and reduced reserve [14,16]. Even when adjusting for age and comorbidities, studies have shown that frailty remains an independent predictor of postoperative complications, mortality, and functional decline [14,16]. This highlights its added value as a complementary measure in perioperative risk stratification.

Additionally, frailty assessment can be incorporated into routine preoperative workflows without increasing time burden. Rapid screening tools such as the CFS or RAI can be completed bedside within seconds to minutes, making them practical in both outpatient and inpatient settings [19]. Early incorporation of these assessments in the preoperative process allows for better identification of patients who may benefit from further evaluation or optimization [19]. There is also growing interest in integrating frailty assessment into electronic medical records. Standardized documentation of frailty scores can improve communication across surgical, anesthesia, and medical teams, and may help guide perioperative planning [19]. In addition, incorporating frailty into pre-existing risk calculators and clinical pathways could further improve its utility in routine practice [19].

Another important role of frailty assessment is in promoting shared decision-making. By providing the patient with a more accurate estimate of surgical risk, clinicians can better counsel them regarding expected outcomes, potential complications, and the chances of prolonged recovery or loss of independence [14,18,19]. This is especially relevant in older adults and high-risk surgical populations, where treatment decisions often involve weighing quality of life against procedural risk [16]. Overall, frailty adds important prognostic information outside of traditional measures such as ASA class, age, and comorbidity burden. As the surgical population continues to age, the routine addition of frailty assessment into perioperative care may improve patient selection, guide optimization strategies, and lead to better outcomes.

Discussion

Key Findings

This review demonstrates that frailty is a strong and independent predictor of adverse outcomes across a broad range of general surgical populations. Unlike chronological age or traditional comorbidity-based risk stratification models, frailty captures a patient’s underlying physiologic reserve and ability to tolerate surgical stress [14,16]. Across elective, emergency, and oncologic surgical settings, frailty has been associated with increased rates of postoperative complications, including SSIs, pneumonia, and prolonged postoperative ileus, as well as higher 30-day mortality [21-26]. In addition, frail patients consistently experience longer hospital lengths of stay, increased rates of intensive care unit utilization, and a greater likelihood of discharge to skilled nursing or rehabilitation facilities rather than home [27-29]. These associations persist even after adjustment for age and comorbidities, underscoring frailty as a distinct and clinically meaningful construct rather than a surrogate for existing risk factors [16]. A summary of these key findings is presented in Table 1.

**Table 1:** Summary of key findings on frailty and surgical outcomes.

Domain	Key finding	Clinical implication
Overall risk stratification	Frailty is a strong independent predictor of adverse surgical outcomes, often outperforming age and comorbidity indices	Should be routinely incorporated into preoperative assessment
Postoperative morbidity	Increased risk of complications, including surgical site infections, pneumonia, and postoperative ileus	Identifies patients who may benefit from preoperative optimization and closer monitoring
Mortality	Associated with higher 30-day postoperative mortality across surgical populations	Enhances prognostic accuracy beyond traditional risk models
Length of stay	Frail patients experience prolonged hospitalization and slower recovery	Anticipate increased resource utilization and plan accordingly
Discharge disposition	Higher likelihood of discharge to skilled nursing or rehabilitation facilities rather than home	Important for discharge planning and patient counseling
Emergency surgery	Frailty is associated with stepwise increases in complications and mortality in emergency general surgery	Supports rapid frailty screening even in acute settings
Oncologic surgery	Frailty correlates with higher complication rates, poorer treatment tolerance, and worse overall outcomes	May influence surgical candidacy and treatment planning
Frailty assessment tools	Tools such as the Clinical Frailty Scale and Risk Analysis Index are practical; phenotype-based tools offer greater physiologic detail	Selection should balance feasibility and accuracy
“Surgical Vital Sign” concept	Frailty provides a global measure of physiologic reserve and vulnerability	Supports integration into routine perioperative workflows

Furthermore, multiple validated frailty assessment tools, including phenotype-based models, clinical judgment scales, and deficit accumulation indices, demonstrate consistent predictive value despite differences in methodology [14,16-20]. Simpler tools such as the CFS and RAI offer rapid bedside applicability, while more comprehensive instruments such as the Fried Frailty Phenotype and EFS provide greater physiologic detail when feasible [15,18,19]. Overall, this evidence supports the conceptualization of frailty as a "surgical vital sign" [14,16].

Limitations of Current Evidence

Despite the growing body of literature supporting frailty as a key determinant of surgical outcomes, several important limitations must be acknowledged. First, there is heterogeneity among frailty assessment tools, with variability in definitions, domains measured, and scoring systems, which limits direct comparison across studies and complicates standardization in clinical practice. Second, there is no universally accepted cutoff or threshold for defining frailty, resulting in inconsistencies in patient classification and risk stratification. Third, most of the existing evidence is derived from retrospective and observational studies, which are inherently subject to selection bias and confounding variables. While these studies demonstrate strong associations, they do not establish causality or determine whether modification of frailty status directly improves outcomes. However, these associations have been consistently observed across multiple independent cohorts and supported by meta-analyses, particularly in relation to increased postoperative morbidity and mortality, strengthening the overall reliability of these findings. Additionally, randomized controlled trials evaluating frailty-targeted perioperative interventions remain limited, restricting the ability to translate predictive insights into evidence-based management strategies. Practical implementation also presents challenges, as time constraints, workflow limitations, and resource availability may hinder routine frailty screening in busy surgical settings. Finally, some frailty tools rely on subjective clinical judgment, introducing interobserver variability that may affect consistency and reproducibility. These limitations highlight the need for more standardized, validated, and scalable approaches to frailty assessment in surgical populations.

Future Directions

Future research should focus on advancing frailty from a predictive marker to a modifiable target within perioperative care. Establishing standardized and universally accepted frailty screening protocols will be essential to facilitate widespread clinical adoption and improve comparability across studies. Integration of frailty metrics into established surgical risk calculators, such as those derived from the American College of Surgeons NSQIP, may enhance predictive accuracy and provide more individualized risk assessments. Emerging technologies, including artificial intelligence and machine learning, offer promising avenues for automated frailty prediction using electronic health record data, potentially enabling real-time risk stratification without additional clinical burden [34]. In parallel, prospective studies and randomized controlled trials are needed to evaluate the effectiveness of frailty-targeted interventions, such as prehabilitation programs, nutritional optimization, and multidisciplinary perioperative care pathways, in improving surgical outcomes. Additionally, greater emphasis should be placed on incorporating frailty assessment into shared decision-making frameworks, particularly in high-risk and elderly populations where treatment decisions must balance procedural benefits with quality-of-life considerations. Ultimately, integrating frailty into routine surgical practice has the potential to transform perioperative care by enabling more precise risk stratification, optimizing patient selection, and improving both clinical outcomes and resource utilization.

## Conclusions

Frailty is a clinically meaningful predictor of surgical outcomes and has been shown to provide prognostic insight beyond chronological age and traditional risk stratification tools. As surgical populations continue to age, the routine assessment of frailty has the potential to transform perioperative care by improving risk prediction, guiding clinical decision-making, and enabling targeted patient optimization. Incorporating frailty into standard preoperative evaluation frameworks may enhance patient selection, facilitate shared decision-making, and ultimately improve postoperative outcomes. Continued efforts toward standardization, integration, and prospective validation are necessary to establish frailty as a fundamental component of modern surgical practice.
